# The Antarctic ozone hole and the pattern effect on climate sensitivity

**DOI:** 10.1073/pnas.2207889119

**Published:** 2022-08-22

**Authors:** Dennis L. Hartmann

**Affiliations:** ^a^Department of Atmospheric Sciences, University of Washington, Seattle, WA 98195-1640

**Keywords:** climate change, ozone hole, climate sensitivity

## Abstract

Since about 1980, the tropical Pacific has been anomalously cold, while the broader tropics have warmed. This has caused anomalous weather in midlatitudes as well as a reduction in the apparent sensitivity of the climate associated with enhanced low-cloud abundance over the cooler waters of the eastern tropical Pacific. Recent modeling work has shown that cooler temperatures over the Southern Ocean around Antarctica can lead to cooler temperatures over the eastern tropical Pacific. Here we suggest that surface wind anomalies associated with the Antarctic ozone hole can cause cooler temperatures over the Southern Ocean that extend into the tropics. We use the short-term variability of the Southern Annular Mode of zonal wind variability to show an association between surface zonal wind variations over the Southern Ocean, cooling over the Southern Ocean, and cooling in the eastern tropical Pacific. This suggests that the cooling of the eastern tropical Pacific may be associated with the onset of the Antarctic ozone hole.

## Significance

We provide a perspective on recent scientific literature that argues the reduction in climate sensitivity identified with relative cooling in the eastern tropical Pacific Ocean could be caused by the onset of the Antarctic ozone hole starting in about 1980. If this is true, the pattern effect could persist as long as the ozone hole, nearly 60 y. This would continue the reduction in warming associated with the pattern effect on climate sensitivity. In addition, increased probability of La Niña events would imply an increased chance of drought in the American Southwest and other impacts of cooling in the eastern tropical Pacific.

Since about 1979, the tropical eastern Pacific Ocean has cooled, while the western tropical Pacific Ocean has warmed. This pattern of sea surface temperature (SST) change has been identified with a reduction in absorbed solar radiation that has slowed the response of global surface temperature to increasing greenhouse gases (1–3). The primary mechanism that relates this SST change pattern to a reduction in apparent climate sensitivity is the development of enhanced boundary layer clouds over the cooler SST region, which reflect more solar radiation and thereby cool the planet. It is important to understand whether this pattern effect is natural variability or part of the forced response to climate change, as this may determine how long the pattern effect will persist and thereby slow the effect of greenhouse gases on global warming. In addition, the pattern of SST trend since 1979 resembles a La Niña event, which has known impacts on seasonal climate around the world, including increasing the probability of drought in the western United States (4). Here we review some recent research on the connection between changes in the Southern Ocean (SO) and changes in the tropical Pacific Ocean. Arguing from connections established in the published literature, we outline a mechanism to connect the reduction in stratospheric ozone in the Antarctic region to cooling of the tropical eastern Pacific. In this mechanism, a surface wind shift in high latitudes associated with the Antarctic ozone hole (5) triggers a cooling of the SO, which, through feedback processes involving atmospheric circulation, low-cloud feedbacks, and ocean current changes, leads to a cooling in the eastern tropical Pacific Ocean. We show, in observations, a connection between stronger winds over the SO, reduced SST there, and associated cooler ocean temperatures in the tropical eastern Pacific Ocean. If this mechanism is what has produced the SST trends since 1980, then we may expect the eastern tropical Pacific to remain relatively cool as the rest of the tropical oceans warm. This would have important consequences for apparent climate sensitivity and for the structure of climate change in the Pacific and for North and South America.

## The Pattern Effect on Climate Sensitivity

**Fig. 1.**
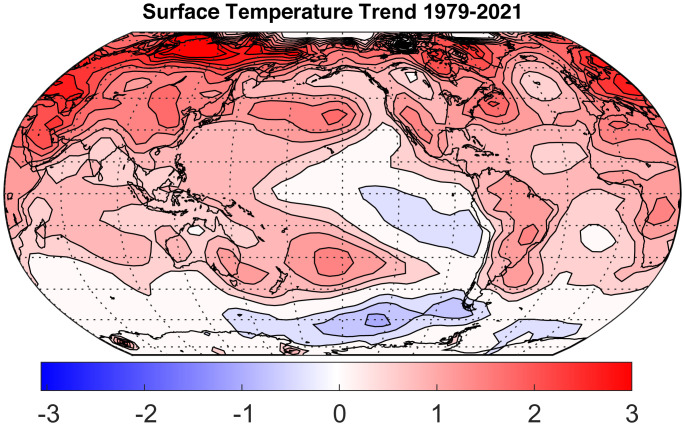
Surface temperature trend from 1979 to 2021 computed from annual means of NOAA data (6). Contour interval is 0.3 ^∘^C; zero contour is not plotted.

Trends in surface temperature since 1979 show a cooling anomaly in the equatorial eastern and southeastern Pacific Ocean, while most of Earth’s surface has warmed. Fig. 1 shows the trend in annual mean surface temperature from 1979 to 2021. It shows net cooling in the eastern tropical Pacific and cooling in the SO, particularly in the Pacific. It has been debated whether the tropical eastern Pacific cooling is natural variability or a response to climate change (7), but it is not a feature of global warming that is simulated by the majority of climate models (8, 9). The majority of climate models produce more warming in the eastern tropical Pacific Ocean than in the western tropical Pacific. One mechanism for the modeled responses is a slowed atmospheric vertical circulation that projects onto the east–west Walker Circulation, which results in weaker easterlies in the tropical Pacific, giving rise to reduced ocean upwelling and warmed SST in the eastern tropical Pacific (10, 11). Recent trends in atmospheric circulation suggest an enhancement in the Walker Circulation in the tropical Pacific rather than a slowing down (12). An alternate possibility more in accord with recent observed trends is that, as the global SST warms, the upwelling of cold water in the eastern equatorial Pacific keeps the surface cool, so that the enhanced SST gradient along the equator may lead to an increased Walker Circulation, leading to enhanced upwelling and cooler surface temperatures in the tropical eastern Pacific (13–16). Some climate models do produce less warming in the eastern Pacific than the western Pacific, in better agreement with observed changes over the past 40 y, and this response appears to be sensitive to small differences in the way the ocean is modeled and how the stratification of the upper ocean is simulated (17, 18). Irrespective of the source of the observed cooling trend in the tropical east Pacific Ocean, it has been shown that the cooling of the eastern tropical Pacific Ocean relative to the overall tropics leads to an increase in low clouds there, which, in turn, causes an enhanced reflection of solar radiation by low clouds that slows the pace of global warming (1–3).

## Recent Work Connecting the SO to the Tropical Pacific

Comparison of climate models to satellite observations showed that clouds over the SO (45^∘^S to 65^∘^S) in climate models reflect less solar radiation back into space than observed clouds, and that this is related to biases in the position of the westerly wind jet over the SO (19). Comparing the bias in SO cloud reflectivity in climate models with the distribution of precipitation in the tropical eastern Pacific showed that models with more reflective clouds in the SO also had improved simulation of the distribution of precipitation in the tropical eastern Pacific (20, 21), giving early indications of how changes over the SO could cause changes in the tropical Pacific. The mechanism proposed was that more reflective clouds in the SO would require more energy transport from the Northern to the Southern Hemisphere, which could be accomplished by moving the tropical precipitation northward, which improves the agreement between modeled and observed precipitation. When this experiment of increasing the reflectivity of SO clouds was conducted in global climate models, however, it was found that, in one model, the required southward energy transport was accomplished by the ocean rather than the atmosphere, so that no improvement in the tropical precipitation pattern was achieved (22). In another model, however, increasing the cloud reflectivity over the SO produced a much more dramatic effect on the tropics, in which the entire eastern tropical Pacific was cooled (23). This work showed that the mechanism for translating the signal of reduced SST over the SO to the tropics hinged upon a strong low-cloud feedback, whereby reduced SST led to increased low clouds, which, in turn, reflected more solar radiation and further cooled the surface, propagating the signal equatorward in the eastern Pacific along the west coast of South America. Prior experiments with a climate model with a slab ocean showed that atmospheric changes over the Southern Pacific Ocean could influence the tropics through a pattern called the South Pacific Meridional Mode (24). Abrupt CO_2_ increase experiments in slab ocean models also show cooling in the SO associated with enhanced oceanic heat uptake in a warmed Earth that is accompanied by cooling in the eastern tropical Pacific (25).

These experiments on the effect of cloud reflection in the SO on climate resulted in a subprogram under the Climate Modeling Intercomparison Project in which a number of different climate models were subjected to the same reduction in absorbed solar radiation over the SO (26). Comparison among these models and with observations showed that models with a strong low-cloud feedback could transmit the cooling signal from the SO to the tropics, and that the models with a stronger low-cloud feedback were in better agreement with observed low-cloud behavior (27). The efficient movement of the cooling signal from the SO to the tropics involves interactions between SST, low clouds, atmospheric circulation, and ocean currents. One of the robust responses in all of these SO cooling experiments is that the zonal winds in the southern Pacific near 60^∘^S increase in response to the cooling. Our hypothesis here is that the wind changes alone that might be forced by the onset of the ozone hole since 1980 could be one cause of the cooling trend in tropical Pacific SST seen in Fig. 1.

## The Connection to the Antarctic Ozone Hole

Between 1979 and about 2000, the westerly winds over the SO intensified (28–30), and the tropical Hadley Cell circulation has expanded to higher latitudes (31). This structure reflects a positive shift in the amplitude of the Southern Annular Mode (SAM) (32, 33). The SAM is a natural mode of variability and so needs only weak forcing to transition to a different average state (34). Research has shown that the trend in the SAM since 1979 is associated with the Antarctic ozone hole, which developed after 1979 and is the principal cause of the observed shift in the zonal mean surface winds over the SO, which has a structure similar to a poleward shift of the SAM (35–37).

**Fig. 2.**
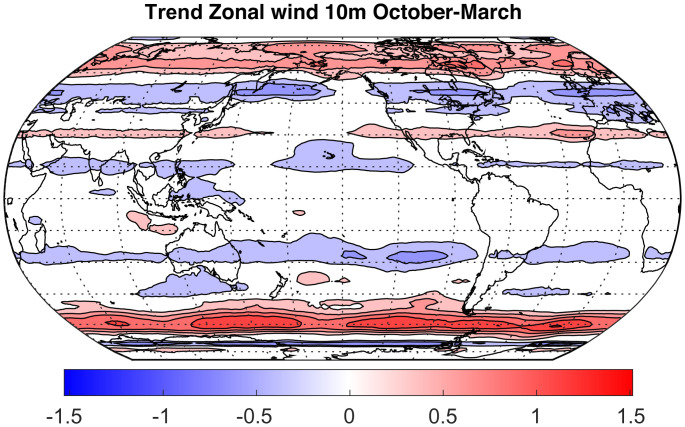
Trend of 10-m zonal wind from 1979 to 2021 computed from October–March means of ERA-5 data (38). Contour interval is 0.2 m ⋅s^−1^; zero contour is not plotted.

Fig. 2 shows the trend in near-surface zonal wind from 1979 to 2021 for the October through March season from ERA-5 reanalysis of observed data (38). Surface westerly winds have increased near 60^∘^S in the summer season since 1979. The zonal wind trend is less significant in the alternative April through September season. The reason for this seasonality of the wind trend is likely twofold. First, the SAM of variability is stronger in the summer season when the climatological winds are more zonally symmetric (39). Second, one might expect the largest response to the Antarctic ozone hole after the spring season, when the ozone depletion is greatest (35, 36). The ozone hole causes the stratospheric westerly vortex to persist longer into the spring season, and these enhanced westerly winds extend to the surface around 60^∘^S. Multiple experiments have shown that ozone depletion over Antarctica can cause an increase in zonal winds near 60^∘^S and a resulting short-term reduction in SST there (40, 41).

The increase in surface winds over the SO induces an enhanced vertical mixing and also equatorward drift of the surface waters that would be expected to cool the surface waters of the SO (42, 43). Analysis of models and data suggest a strong connection between wind trends and the cooling SSTs in the SO (41). Stronger zonal winds also enhance sea ice extent and may explain why Antarctic sea ice has remained relatively constant while Arctic sea ice has declined in recent years (44–46). Although the connection between winds and sea ice is weaker than the connection to SST (47), anomalously weak westerly winds are believed to have contributed to rapid reductions of sea ice in 2016 (48).

Gravity observations have been used to show that increased zonal winds at 60^∘^S associated with the SAM are accompanied by an increased water mass in the southeast Pacific, which has, associated with it, a northward geostrophic current off the west coast of Chile (49). This northward current would carry cold water toward the north, feeding into the Peru Current. This northward advection of cold water would cause enhanced low-level clouds which extend the cooling poleward (23, 27). The increased zonal winds at 60^∘^S coupled with surface cooling extending farther into the subtropics lead to anomalous high pressure in the atmosphere off the coast of South America, which results in enhancement of the equatorward winds in the southeast Pacific, which advect the cooling equatorward and may also enhance the Peru Current upwelling as well as easterly winds closer to the equator. These changes lead to cooling in the eastern tropical Pacific Ocean (27, 50, 51).

Bjerknes feedback, whereby an increased westward SST gradient at the equator leads to increased easterlies, which induce increased upwelling and further cooling along the equator in the eastern equatorial Pacific, completes the set of coupled interactions that lead from enhanced westerlies at 60^∘^S to cooling in the eastern tropical Pacific. In the next section, we will use monthly observations of zonal wind and SST to look at the observed relationship between wind variations over the SO associated with the SAM and SST.

## The SAM and SST

To investigate the relationship of the SAM wind variations to SST, we form an index of the SAM that is the difference in 10-m winds averaged over 55^∘^S to 65^∘^S minus the zonal-mean winds averaged over 35^∘^S to 45^∘^S. A zonal wind index is used rather than the more traditional SAM index based on sea level pressure (28, 29), because our hypothesis links SST to zonal wind, not to surface pressure. The times when this SAM index is ±1 SD away from its mean value are used to composite the SST. The SAM index is weakly negatively correlated with the Niño 3.4 index of tropical SST variability, which indicates that La Niña events are correlated with enhanced westerlies at 60^∘^S. This is consistent with our hypothesis, but it is also consistent with a hypothesis that La Niña events drive SAM events (52). El Niño–Southern Oscillation (ENSO) is known to initiate anomalies that propagate from the tropical western Pacific into the SO (53–56), where they can influence the SAM. We remove linear trends from the SST and the SAM index prior to analysis. To minimize the effect of ENSO on the response and isolate the SAM effect, we also remove that part of the SST variance that is linearly associated with the Niño 3.4 index and that part of the SAM index time series that is correlated with the Niño 3.4 index of ENSO. After these modifications are made, we perform the composite difference of SST between SAM positive and negative events. In this way, we hope to remove the influence of ENSO on SAM, and isolate month-to-month connections between SAM and SST that are independent of ENSO.

**Fig. 3.**
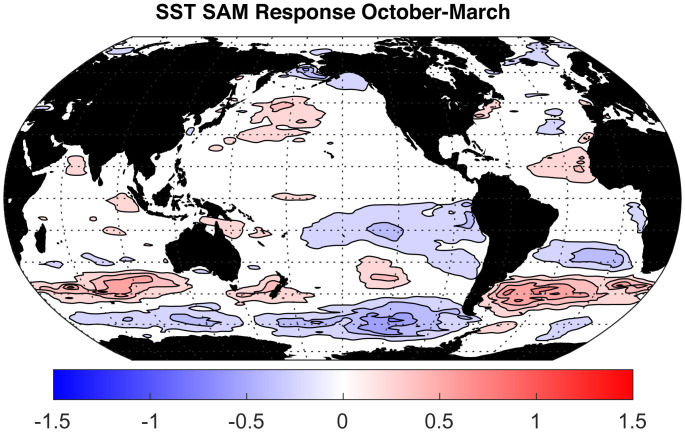
Response of October–March monthly SST to the monthly SAM index, based on ERA-5 data (38). Contour interval is 0.15 K; zero contour is not plotted.

The result of this compositing analysis is shown in Fig. 3, which indicates that the SST cools in the southern Indian and Pacific Oceans in response to positive surface wind anomalies at 60^∘^S, particularly strongly west of the Drake Passage in the southern Pacific, in agreement with prior analyses (41). In addition to these expected responses in high latitudes, cooling of SST is shown in the tropical eastern Pacific south of the equator. Note that the northern Pacific shows no significant SST anomalies, indicating that the expected ENSO signature there has been effectively removed by regressing out the linear dependence on Niño 3.4. SST anomalies in the Southern Hemisphere, including the response in the tropical Pacific, are significantly different from zero at *P* = 0.01 using a simple *t* test. In addition, we did the same analysis using a different wind and SST analysis, which produced similar patterns (*SI Appendix*). This SST response is similar in shape to the SST response found in the SO cooling experiments (23, 27). These results are subject to uncertainty, and the mechanisms need to be investigated in a series of carefully controlled modeling experiments, but the weight of evidence from observations and model experiments suggests that some significant part of the cooling in the eastern tropical Pacific may be triggered by westerly wind accelerations associated with the Antarctic ozone hole.

## Summary and Conclusion

Recent modeling work has shown that cooling of the SO can lead to cooling of the tropical eastern Pacific. The mechanism involves low-cloud feedbacks, atmospheric circulation, and ocean current responses, which are some of the more uncertain aspects of climate models. Westerly surface wind intensification near 60^∘^S leads to cooling of the SO. The Antarctic ozone hole is known to have caused increases in surface zonal winds at 60^∘^S. Observed responses of SST to zonal wind variations at 60^∘^S show a cooling signal in the eastern tropical Pacific. It is therefore plausible that the cooling in the eastern tropical Pacific that has been observed since 1980 is associated with the development of the Antarctic ozone hole.

Several alternative explanations for the cooling of the SO and eastern tropical Pacific Ocean are possible. It has been proposed that freshwater input to the SO from melting of Antarctic ice sheets and shelves could lead to cooling of the SO (57), especially as the SO continues to warm. Cooling of the tropical and southern Pacific Ocean could be related to natural decadal variability of the ocean–atmosphere system (52, 58). It is possible that our removal of the effect of ENSO by linear regression does not entirely remove the effect of ENSO on the southern Pacific Ocean. Nonetheless, evidence from both models and data indicates that wind and SST anomalies over the SO can influence the tropics.

International regulation to limit release of ozone-destroying gases appears to have led to some initial signs of ozone hole healing (5), but the Antarctic ozone hole is expected to persist for another 50 y. Therefore, we may expect its effect on SST to persist. Since human-induced global warming is also expected to drive a poleward shift of the westerlies over the SO (59, 60), the current jet shift and its SST signature may persist even longer than the ozone hole, depending on the persistence of the ozone hole and the rate of global warming (61, 62). This shift of tropical SST also implies a greater frequency or intensity of La Niña events, which have a known effect on North American weather, including a greater likelihood of droughts in the American Southwest (4).

## Methods

The surface temperature data in Fig. 1 were obtained from National Oceanic and Atmospheric Administration (NOAA) Global Surface Temperature (NOAAGlobalTemp) data provided by the NOAA/Oceanic and Atmospheric Research (OAR)/Earth Systems Research Laboratory (ESRL) PSL, Boulder, CO (6) from their web site at https://www.ncei.noaa.gov/access/metadata/landing-page/bin/iso?id=gov.noaa.ncdc:C00934 (63). Annual mean anomalies were used, and the trend was calculated by linear regression.

The zonal wind and SST data shown in Figs. 2 and 3 were obtained from the ER5 reanalysis dataset (38) at https://cds.climate.copernicus.eu/cdsapp#!/dataset/reanalysis-era5-single-levels-monthly-means?tab=form (64).

Indices of Niño 3.4 were obtained from https://www.cpc.ncep.noaa.gov/data/indices/ersst5.nino.mth.91-20.ascii (65). The Niño 3.4 monthly time series was used to remove the Niño 3.4 signal from the SAM index and from the wind and SST data sets by linear regression. This assures that the composites we show in 3 have no linear relationship to Niño 3.4. Significance of the differences in SST between SAM positive and SAM negative events was tested with a t-statistic and found to be significant at *P* = 0.01 (*SI Appendix*).

## Supplementary Material

Supplementary File

## Data Availability

All study data are included in the article and/or *SI Appendix*. Software used and some intermediate data sets are provided at the University of Washington ResearchWorks Atchive (http://hdl.handle.net/1773/49168) (66).
