# Supplementary material for: The Antarctic ozone hole and the pattern effect on climate sensitivity
Source: Proc Natl Acad Sci U S A. 2022 Aug 22;119(35):e2207889119. doi: 10.1073/pnas.2207889119 (PMC9436325; doi:10.1073/pnas.2207889119)
Supplement: Supplementary File [file pnas.2207889119.sapp.pdf]

Supplementary Information:
The Antarctic Ozone Hole and the pattern effect on climate sensitivity
By D.L. Hartmann

First we show the statistical significance of the composite differences in Figure 3. Colored contours show the regions where the differences in Fig. 3 are significant at the p=0.01 and p=0.001 levels, using a standard t-test.

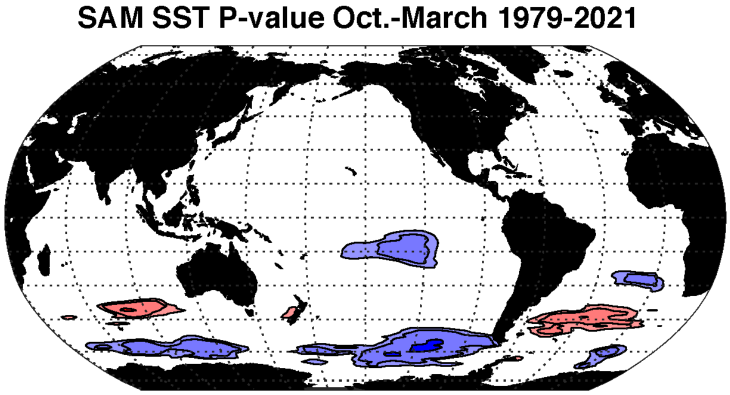

Figure S1 Statistical significance of the SST differences in Figure 3. Colored areas show where the differences in Fig. 3 exceed the p=0.05 *a priori* significance level (light blue or red) and where they exceed the p=0.01 significance level (darker blue or red). These are based on the variance of the SST after the trend and Niño 3.4 signals have been removed.

Next we show the trend in zonal wind at 10 meters from ERA-5 reanalysis for the October-January season to compare with Figure 2, which shows the trend for the October to March season. The point is that the trend shows more of a SAM-like signature, with increased winds at 60˚S and decreased zonal wind at 40˚S.

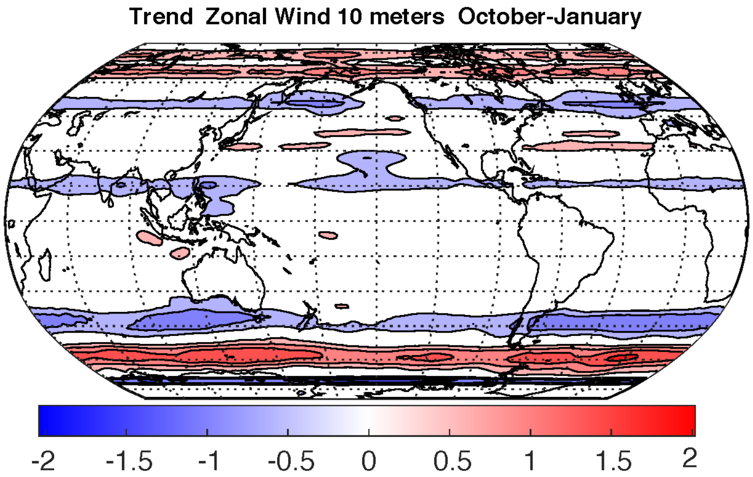

Figure S2: Zonal wind at 10 meters trend from 1979 to 2021 for the October through January season.

With the extension of the ERA5 reanalysis data set back to 1959 we were able to compute the 10-meter zonal wind trends for 40-year periods from 1959-2001, 1969-2011 and 1979-2021. In each case a similar trend was found for the October-March season. The wind shift mostly occurred in 1979-2001, so all these 40-year periods include the wind shift associated with the ozone hole onset, and estimate it similarly.

We repeated the analysis, except using NCAR/NCEP reanalysis for the surface zonal wind index and NOAA ERSST analysis of SST. We repeated the analysis for the 1950-2021 period, and performed the analysis separately for the 1950-1985 and 1986-2021 periods. In addition, rather than using the difference in zonal mean wind difference between 55-65˚S and 35-45˚S, we use the first principal component of zonal wind between 35˚S and 75˚S. The results are shown in Fig. S3. Figure S3 shows that similar negative SST departures are obtained for this different data set and for the two sub periods of 1950-1985 and 1986-2021.

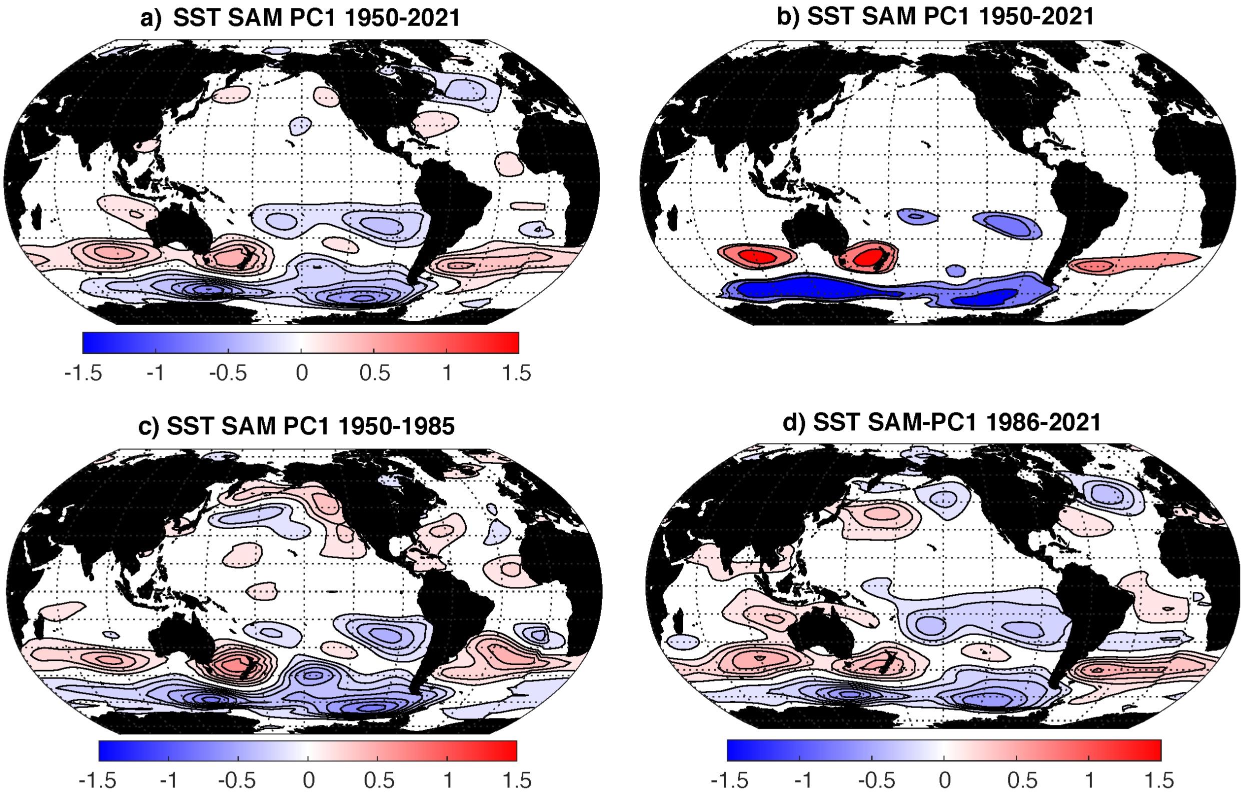

Fig. S3 Composites of the SST difference between cases when the first principal component of surface zonal wind is greater or less than its mean value by one standard deviation. A) for the 1950-2021 period, b) the p-value where values less than p=0.05 are shaded. c) same as panel a except for 1950-1985 and d) 1986-2021.

We computed the SST composites as before for the 1959-2021 period using the ERA5 analyses of SST and zonal wind at 10 meters. In addition we lagged the SST fields relative to the zonal wind index. Figure S4 shows the composited zonal wind anomalies at zero lag in panel a and the SST composites relative to the wind index at lags of 0 (b) -1 (c) and +1 (d) months. These

show that there is a signal in the SST prior to the month in which the zonal wind index sets a key date for compositing. This is unexpected if the wind field is driving the SST anomalies, since the zonal wind anomaly in the month corresponding to the lag -1 SST is small. In the month following the zonal wind anomaly (lag +1, panel d) the tropical SST anomaly is larger and has propagated toward the east and south. The zonal wind anomaly barely persists at lag +1, and what anomaly remains is in the far eastern South Pacific (Fig. S4 panels e and f). While the pre-appearance of an SST anomaly is unexpected, the greater SST anomaly after than before the wind anomaly is suggests that the wind anomaly causes the SST anomaly. The SST has an intrinsically longer time scale than the zonal wind index.

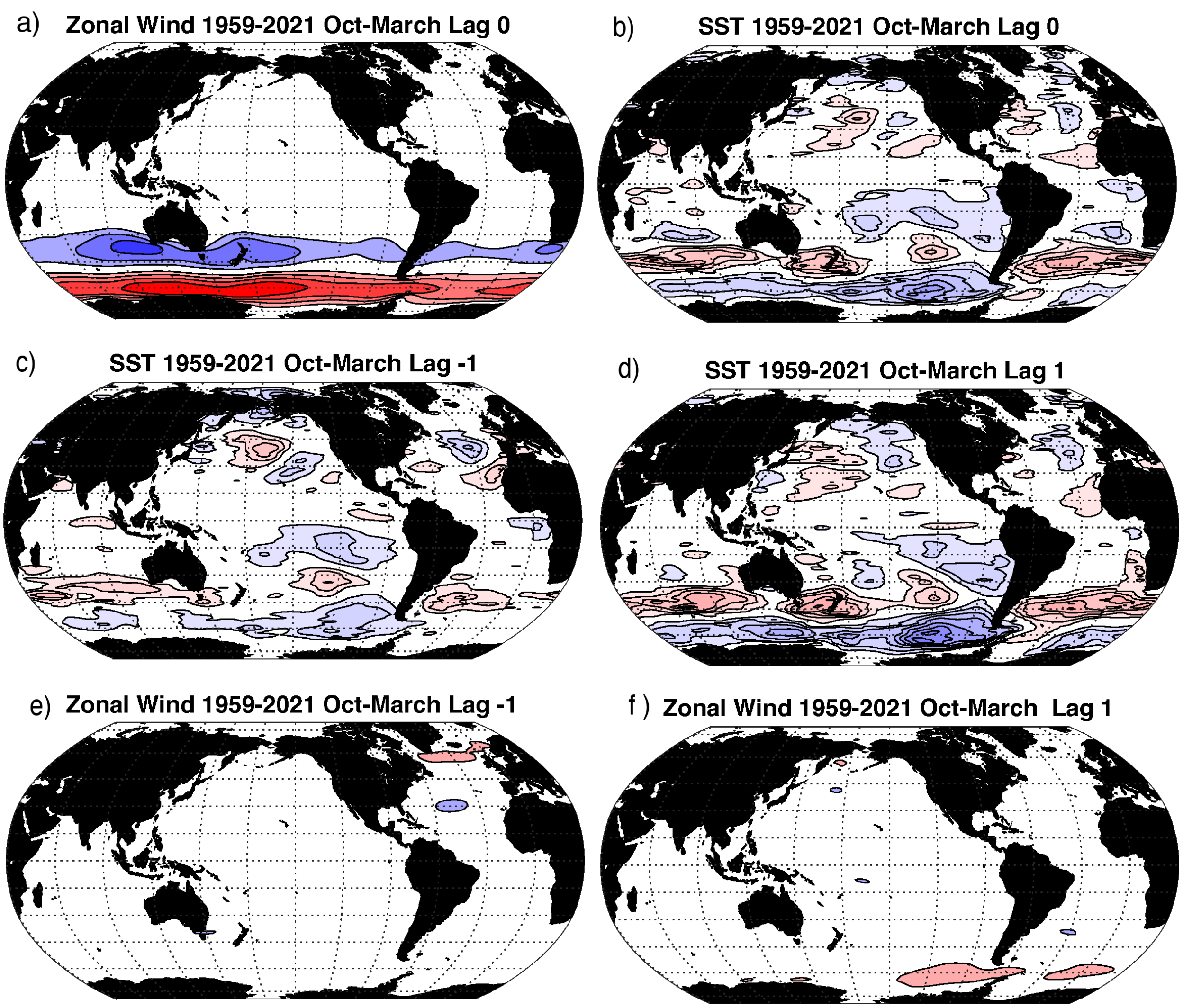

Figure S4 a) Composites of Zonal wind at 10-meters from ERA5 reanalysis for the period 1959-2021, b) SST anomaly corresponding to panel a), c) and d) same as panel b), except one month prior (Lag -1) and one month after (Lag +1) the time of the wind anomaly shown in a). Zonal wind anomalies the month before e) and the month after f} the time shown in panel a). Contour interval is 1 ms^{-1} for zonal wind and 0.1K for SST, zero contour not plotted.

Data access.

NCEP/NCAR Reanalysis surface winds.
https://psl.noaa.gov/data/gridded/data.ncep.reanalysis.pressure.html

NOAA Extended Reconstructed Sea Surface Temprature (SST) version 5
https://psl.noaa.gov/data/gridded/data.noaa.ersst.v5.html

ERA5 Reanalysis
https://cds.climate.copernicus.eu/#!/search?text=ERA5&type=dataset
